# Screening Guidelines and Programs for Cervical Cancer Control in Countries of Different Economic Groups: A Narrative Review

**DOI:** 10.7759/cureus.41098

**Published:** 2023-06-28

**Authors:** Jyoti Sharma, Madhavi Yennapu, Yamini Priyanka

**Affiliations:** 1 Council of Scientific and Industrial Research-National Institute of Science Communication and Policy Research (CSIR-NIScPR), Academy of Scientific and Innovative Research (AcSIR), New Delhi, IND; 2 Division of Non-Communicable Diseases, Indian Council of Medical Research, New Delhi, IND; 3 Division of Reproductive Child Health and Nutrition, Indian Council of Medical Research, New Delhi, IND

**Keywords:** health system challenges, implementation, barriers, program, guidelines, screening, cervical cancer

## Abstract

Screening guidelines and practices differ according to resource availability and continually update as scientific developments take place. In this article, we have reviewed screening guidelines and programs for cervical cancer prevention in selected countries belonging to different economic groups viz high income, middle income, and low income. We have selected six countries - the United States of America (USA), the United Kingdom (UK), India, South Africa, Bangladesh, and Malawi. Considerable differences are observed across the health systems. Countries with established screening guidelines complemented by organised nationwide programs or insurance practices have much better screening rates. Human Papilloma Virus (HPV) DNA testing is currently the test of choice in the majority of settings for cervical cancer screening due to its higher sensitivity (up to 90-100%) and longer screening intervals (three to five years). It is also cost-effective, less dependent on operator expertise, and suitable for all settings as compared to a Pap smear test or visual inspection with acetic acid (VIA). Self-sampling of HPV can further help to improve screening coverage by increasing opportunities of reaching to women who would otherwise not participate in screening programs. Resource-constrained countries recommend VIA-based screening in their national programs due to its low cost. The share of cervical cancer is higher in middle and low-income countries as they have lower screening coverage, compared to high-income countries. The main barriers faced in the implementation of the program in low-income countries (LICs) are pertaining to the health system, patient-specific challenges, and healthcare provider-specific challenges.

## Introduction and background

The fourth most common cancer amongst women across the globe in terms of the number of new cases and deaths is cervical cancer after breast, colorectal and lung [1]. According to Global Cancer Observatory (GLOBOCON) data, 604127 new cases of cervical cancer and 341831 deaths were reported in females in 2020 [1]. Cervical cancer is a preventable cancer and is linked to the socioeconomic status of women. The highest incidence rates of cervical cancer are found in low- and middle-income countries (LMICs) with age-standardised incidence rate (ASIR) of 23.8 in LICs and 16.9 in LMICs [1] (Table 1) and particularly in sub-Saharan Africa [2]. High-income countries have less burden of cervical cancer due to well-placed screening programs. However, regions of high socioeconomic status are also not immune to Human Papilloma Virus (HPV) infections and associated malignancies [3]. Almost 90% of cervical cancer deaths occur in resource-constrained countries and affect the poorest and most vulnerable women disproportionately through their child-bearing and economically productive years [4]. GLOBOCON 2020 data says that cervical cancer is the top cancer site in females in 23 countries after breast cancer which is the top cancer site in 160 countries [1]. It is caused mainly by two strains of HPV-16 and HPV-18 [5]. GLOBOCON data shows that some regions are at a higher risk of cervical cancer than others. The African region has the highest cervical cancer burden [1]. The ASIR in African regions is as high as 82.4 in Eswatini and 68.2 in Malawi. In Asia, Maldives and Indonesia have the highest ASIR (24.1) and 23.7 respectively for cervical cancer. Montenegro with ASIR 26.4 and Romania with ASIR 22.5 have the highest cervical cancer rates in Europe. The USA has an ASIR of 6.2 and Canada has an ASIR of 5.5 in North America. Bolivia and Paraguay have ASIR of 36.6 and 32.5 respectively in South America. In Oceania, Fiji (27.8) and Papua New Guinea (27.7) have the highest ASIRs.

**Table 1:** Summary statistics of cervical cancer in 2020 in countries based on economy Compiled from GLOBOCAN 2020 Abbreviations: LICs: low-income countries LMICs: low-middle-income countries UMICs: upper-middle-income countries HICs: high-income countries ASIR: Age-Standardized Incidence Rate

Income group	ASIR	Rank of cervical cancer (new cases)	Top 5 most frequent cancers in females
LICs	23.8	2^nd^	Breast, Cervix uteri, Colorectum, Lung, Ovary
LMICs	16.9	2^nd^	Breast, Cervix uteri, Colorectum, Ovary, Lung
UMICs	12.8	10^th^	Breast, Colorectum, Lung, Thyroid, Cervix uteri
HICs	NA	21^st^	Breast, Colorectum, Lung, Corpus uteri, Thyroid

There are two ways of avoiding cervical cancer - primary prevention and secondary prevention. Primary prevention prevents precancers in the first place using HPV vaccination. Secondary prevention detects precancerous lesions by screening and treating them before they turn into cancer [6]. Developed countries have seen a significant decline in cervical cancer-related mortality [7] and this has been associated with the application of organised cervical screening programs [8]. Screening for cervical cancer is logistically a complex and resource-intensive process [9] Three most commonly practised approaches exist to screen for cervical cancer, each designed for a particular socio-economic stratum viz VIA, cytology/Papanicolaou (Pap) smear test and HPV DNA testing [9]. The coverage rate of the current cervical cancer screening program varies depending on a country’s profile. While 81% of countries have cervical cancer screening policies and strategies, only 48% have an operational plan with funding [10]. For every country, there exists a different set of challenges in implementation. Since screening and treatment can be done using different primary screening and triage tests, there are numerous possible combinations or algorithms [11]. Developed countries have institutionalised Pap cytology tests or HPV DNA testing as the primary method of screening. However, the same modalities cannot be adopted for resource-constrained settings as there are various operational challenges which include factors related to the type of test, factors related to logistics, infrastructure and human resources, factors related to the target population covered, and factors related to program design [9]. However, despite the availability of a variety of screening tests, it is program implementation and organisation that determines the success of cervical cancer screening in a country as quoted by Bhatla et al. [12].

Research question

How do screening guidelines for cervical cancer get shaped according to resource availability and what are the national programs for cervical cancer control in the selected countries and what barriers are faced in implementation of the programs?

This review aims to identify and summarise cervical cancer screening guidelines and national screening programs in the selected countries of different economic groups viz high-income countries (HICs), middle-income countries (MICs) and low-income countries (LICs). Barriers to implementation of the programs are also reviewed which would help policymakers of low-coverage countries to identify the bottlenecks. Evidence from literature was collated and various guidelines and programs in different countries have been summarised. Incidence, mortality, and screening-rate data were obtained from GLOBOCAN and World Health Organization (WHO) websites and tabled.

## Review

Selection of countries

The following selection criteria were used for the selection of countries. (1) Countries from each group viz HICs, LMICs and LICs should have established guidelines and screening systems in place. (2) Information should be available on screening programs. (3) Burden of cervical cancer should be significant. (4) From each income group defined by World Bank, we have restricted our study to two countries. In HICs, the USA was taken as it is the only developed country which does not provide universal healthcare to its citizens but still has achieved high screening rates. On the contrary, the UK has an extremely good government-funded health system in place. So it would be interesting to see how screening rates get affected due to differences in health systems. In MICs, we chose India because it is the country where the authors are situated. South Africa was chosen as it has high HIV rates and we wanted to see how cervical screening guidelines get shaped due to HIV status as HPV and HIV have a synergistic relationship. In LICs, we chose Malawi because it has the second-highest ASIR for cervical cancer across the globe. Eswatini has the highest ASIR but this country has a small population and the number of cervical cancer cases is very less, so it was not selected for this review. Bangladesh was chosen because despite being less developed it has a good health system and is on the path to graduating from the UN’s Least Developed Countries (LDC) list by 2026. It was one of the first countries to implement VIA-based screening for cervical cancer.

Burden of cervical cancer in the selected countries

Table 2 summarises the burden of cervical cancer according to GLOBOCAN 2020 [1] in selected countries of this review. Cervical cancer ranks first in Malawi in terms of the number of new cases reported with an ASIR of 68.2 per 100,000 women and third in India (ASIR 16.8 per 100,000) and South Africa (ASIR 33.4 per 100,000). The USA (ASIR 6.2 per 100,000) and the UK (ASIR 9.9 per 100,000) have a lower burden of cervical cancer and it ranks 21st in both countries. Bangladesh has an ASIR of 10.6 per 100,000 women and cervical cancer is the fifth most common cancer in Bangladesh's population.

**Table 2:** Region-specific incidence and mortality rates for cancer of the cervix in 2020 (Compiled from GLOBOCON 2020) Abbreviations: USA: United State of America UK: United Kingdom ASR: Age Standardized Rate

Country	New cases (No.)			New cases (%) of all cancers	Rank (new cases)	Deaths (No.)	Deaths (%) of all cancers	ASR (Incidence) per 100,000 women	ASR (Mortality) per 100,000 women
USA	13545		0.59		21	5706	0.93%	6.2	2.0
UK	3791		0.83		21	1121	0.62%	9.9	1.8
India	123907		9.4		3	77348	9.1%	16.8	10.2
South Africa	10702	9.9			3	5870	10.3%	33.4	18.3
Bangladesh	8268	5.3			5	4971	4.6%	10.6	6.4
Malawi	4145	23.1			1	2905	23.3%	68.2	51.2

Screening guidelines, systems and national programs in selected countries

We have extracted the most recent guidelines for each country in the scope of our review as well as those that preceded them in order to have an overview of the changes made in guidelines over time. In addition, we also searched relevant grey literature on policy documents from the Ministry of Health (MoH) websites of the included countries. Globally issued recommendations for cervical cancer screening by WHO have complimented our extraction of national guidelines.

WHO guidelines

For the general population of women, WHO’s recent 2021 guidelines now recommend screening with HPV DNA as the primary test starting at age 30 at intervals of five to ten years instead of Pap Smear or VIA [13]. Self-sampling is another option that is suggested by WHO. Specifications for women living with HIV (WLHIV) have also been given [14]. Among the 23 recommendations, six are identical for both the general population of women and for women living with HIV and 12 are different and specific for each population. For women living with HIV, HPV DNA testing has been recommended as a primary screening test starting at the age of 25 years at intervals of three to five years [13]. HPV DNA testing has higher sensitivity (90 to 100%) [15,16] compared to pap cytology and VIA. It is also more cost-effective than visual inspection techniques or cytology and suitable for all settings [17].

High-income countries

USA

There are five main agencies identified for formulating guidelines for cervical cancer in the USA. These are the American Cancer Society (ACS), U.S. Preventive Services Task Force (USPSTF), American Congress of Obstetricians and Gynecologists (ACOG) and American Society for Colposcopy and Cervical Pathology (ASCCP) and American Society for Clinical Pathology (ASCP), and Society of Gynecologic Oncology (SGO) [18]. The agencies keep releasing Practice Bulletin and Practice Advisory for minor updates at regular intervals. The initiation and cessation age of screening and the type of test to be followed varies according to the recommending authority. The latest ACS 2020 guidelines do not recommend screening for the age group 21-24 and screening should begin at 25 years. This change is mainly related to HPV vaccines. The first cohort of women who received the HPV vaccine at a younger age has led to increasing the age of starting screening to 25 years as the vaccine prevents the development of cervical precancer. HPV testing every five years is currently the primary test of choice instead of a pap smear [19,20]. USPSTF 2018 recommend a pap test every three years (for the 21-29 years age group). For the 30-65 age group HPV test or HPV/pap co-test every five years is the recommended test.

Previously ACS (2012), USPSTF (2012), and the ACOG (2013), all recommended that women should begin participating in cervical cancer screening at 21 years of age and that subsequent screening should occur every three years until age 29. For women aged 30 to 65 years, if HPV testing is used in conjunction with Pap smears the screening interval may be extended to every five years [21]. Screening is not recommended above 65 years if continuous negative test results are obtained.

The US has no nationwide population-based screening program in place. Screening is opportunistic and is largely the responsibility of individual private practitioners functioning in the context of public/private health insurance plans [22]. Despite this, the US has achieved the highest screening rates.

UK

The UK has a highly successful organised population-based screening program. The screening was introduced in a background of opportunistic screening during a period of time when the risk of disease was increasing. National Health Service Cervical Screening Programme (NHSCSP) started in 1988 [23] and has helped in bringing down cervical cancer-related mortality. NHSCSP guidelines replaced the Papanicolau smear with liquid-based cytology in 2000 on recommendation by National Institute for Health and Care Excellence (NICE) [24] and altered the commencement of screening at the later age of 25 instead of 20 [25]. A large number of European countries including the UK are now considering HPV testing as the primary screening test [26,27]. Currently, in England, women aged 25-64 years are sent screening invitations by mail under the program. Eligible women at the age of 24.5 years receive the first invitation. Women aged 25 to 49 years receive invitations every three years and women aged 50 to 64 years receive invitations every five years [28].

Middle-income countries

India

India, like other developing countries, is low in resource settings and is facing a number of challenges in the management of cervical cancer. Operational guidelines for the management of common cancers in India were launched in 2016 by the MoH. It recommends screening with VIA every five years [29]. However, the on-field implementation of cervical cancer screening on a national scale is still a herculean task [11]. The Federation of Obstetrics and Gynaecologic Societies of India (FOGSI) guidelines [30] made resource-stratified recommendations for the first time in 2018. There are marked variations in resources in India for cervical cancer screening. FOGSI guidelines stratify the health system into two resource settings - good or limited and describe the mode of screening and treatment for each. HPV testing is the recommended test of choice for cervical cancer screening. VIA is recommended for low-resource settings until an affordable HPV test becomes available. The National Cancer Grid [31] released its Consensus Evidence Based Resource Stratified Guidelines on Secondary prevention of Cervical, Breast & Oral Cancers in 2019 and has given recommendations according to resource availability viz essential/limited resource, optimal/enhanced resource and optional/high resource. It has recommended VIA for low resource settings (for women aged 30-65 years; one to three times in a lifetime), HPV testing for enhanced (for women aged 30-65 years; at 10-year intervals) and high resource settings (for women aged 25-65 years; at five-year interval) [31]. The country has been implementing the National Program for Control of Cancer, Diabetes, CVDs and Stroke (NPCDCS) since 2010 under the National Health Mission for screening of common cancers [32]. The program got renamed as National Program for Prevention and Control of Non-Communicable Diseases (NP-NCD) in May 2023 [33].

South Africa

In South Africa, HIV rates are high due to which cervical screening guidelines have specified recommendations based on HIV status. At present, the national cervical cancer prevention programme in South Africa offers three cervical cytology smears per lifetime, starting after the age of 30 at 10-year intervals [34]. No end age has been mentioned for screening. In the private sector, cytology-based opportunistic screening is well accepted but not uniformly implemented. Guidelines for patients with HIV infection include more frequent cytology tests [35]. A systematic review by [36] concluded that HIV-positive women are at an increased risk of developing cervical cancer than HIV-negative women. This may partly be due to HIV's modifying effect on HPV pathogenesis. So it is important that they are screened early and often [2]. WHO has also quoted that women living with HIV have six times more likelihood of developing cervical cancer compared to women without HIV. Cervical cytology and HPV testing are both considered suitable for screening in South Africa and screening practitioners or facilities should choose the most appropriate test for their setting. HPV-based screening is preferred to cytology because the higher sensitivity allows a longer safe interval, but both tests remain valid options [35].

Low-income countries

Malawi

Malawi has the second highest cervical cancer incidence and mortality in the world with ASIR of 68.2 and 51.2 per 100,000 population respectively [1]. In response, the MoH established a cervical cancer screening programme using VIA and treatment of precancerous lesions with cryotherapy in 2004 for women aged 30-45 years in Mulanje district as a pilot project [37]. It recommends screening once every five years for HIV-negative women and once every 2-3 years for HIV-positive women. It is one of the first countries in eastern and southern Africa to introduce VIA based screen and treat programme nationally. The programme has now expanded to all districts. The National Cervical Cancer Control Strategy for 2016-2020 by MoH, Malawi recommends screening with VIA (25-49) age group and outlines comprehensive interventions, including the integration of cervical cancer screening services into HIV care [38].

Bangladesh

Cervical cancer is the second most common cancer in Bangladesh. A national cervical cancer screening programme was launched in 2004 using visual inspection after the application of acetic acid (VIA) as the screening test [39]. It is one of the first countries to introduce VIA for the national programme. The National Strategy for Cervical Cancer Prevention and Control in Bangladesh 2017-2022 released by the Government of Bangladesh with technical support from the Bangabandhu Sheikh Mujib Medical University (BSMMU), the World Health Organization (WHO) and the United Nations Population Fund (UNFPA) in 2017 targets to screen women aged 30-60 years every 5 years with VIA [40]. Guidelines by the Bangladesh Society for Colposcopy & Cervical Pathology (BDSCCP) recommend screening age from 21 to 60 years. Different intervals have been recommended for different age groups with VIA/PAP [41].

**Figure 1:**
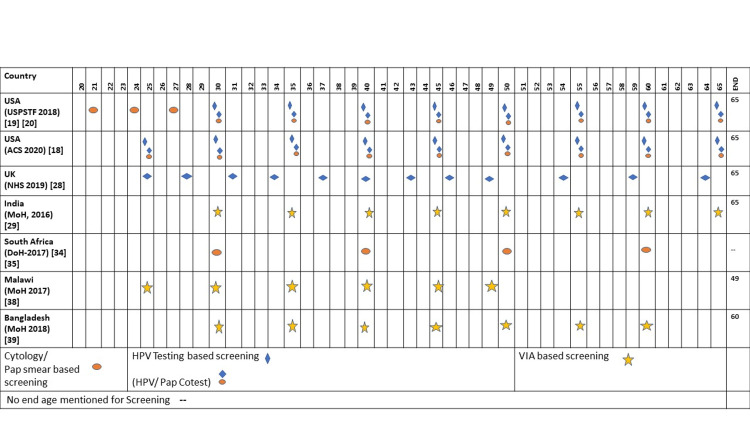
Screening intervals and primary recommended tests in national programs/guidelines of different countries Source: Compiled from multiple sources Abbreviations: U.S. Preventive Services Task Force (USPSTF) American Cancer Society (ACS) National Health Service-Cervical Screening Programme (NHS-CSP) Ministry of Health (MoH) DoH (Department of Health)

Table 3 and Figure 1 summarise cervical cancer screening practices in the selected countries and screening intervals in national programs/guidelines respectively.

**Table 3:** Summary of cervical cancer screening practices in selected countries Source: Compiled from multiple sources ^a^ WHO-Cervical Cancer Country Profiles, 2021 (https://www.who.int/publications/m/item/cervical-cancer-country-profiles) Abbreviations: American Cancer Society (ACS) U.S. Preventive Services Task Force (USPSTF) National Health Service Cervical Screening Programme (NHS-CSP) Ministry of Health (MoH) Federation of Obstetrics and Gynaecologic Societies of India (FOGSI) National Cancer Grid (NCG) National Program for Control of Cancer, Diabetes, CVDs and Stroke (NPCDCS) National Programme for Prevention and Control of Non-Communicable Diseases (NP-NCD) Department of Health (DoH) Cervical Cancer Control Programme (CECAP) Bangladesh Society for Colposcopy and Cervical Pathology (BDSCCP)

Country	Name of agencies formulating guidelines	Nationwide screening program	Beginning of the program	Recommended primary screening method	Targeted age	Interval	Screening coverage (ever screened)
USA [20]	ACS 2020	No	na	HPV testing	25-65 (ACS 2020)	5 years	88% ^a^
UK [23,28]	NHS-CSP	Yes, National Health Service Cervical Screening Programme (NHSCSP)	1988	HPV Testing	25-65	25 to 49 (3 years) 50 to 65 (5 years)	89% ^a^
India [29,30-33]	MoH-2016 FOGSI-2018 NCG-2019	Yes National Program for control of Cancer, Diabetes, CVDs and Stroke (NPCDCS). Renamed as NP-NCD in May 2023 but partially functional	2010	VIA	30-65	5 years	2% ^a^
South Africa [2,34]	National Guideline for Cervical Cancer Screening Programme by DOH	Yes National Cervical Cancer Prevention Programme	2001	HPV Testing And Cytology	30 with no upper age limit	10 y	52% ^a^
Malawi [37,38]	The National Cervical Cancer Control Strategy (2016-2020) by MoH	Yes Cervical Cancer Control Programme (CECAP)	2004	VIA	25-49 years^a ^(MoH) 30-45 years [37]	5 y for HIV neg women 2-3 y for HIV-positive women	19% ^a^
Bangladesh [39,40,41]	National Strategy for Cervical Cancer Prevention and Control in Bangladesh, 2017-2022 BDSCCP-2019	Yes National Cervical Cancer Control Program	2004	VIA	30-60	5	7% ^a^

Screening coverage/rates

The WHO’s call for the elimination of cervical cancer targets to achieve a 70% screening coverage rate by 2030 [13]. Data from WHO’s cervical cancer country profiles [42] depict that there are large differences in screening coverage of the countries of different economic groups (Table 2). Effective rates are observed in high-income group countries with established guidelines - USA and UK which have rates of 88% and 89% respectively. Cervical cancer incidence rates declined significantly in the US after the implementation of the Papanicolaou (Pap) smear [21]. In South Africa, 52% ever got screened for cervical cancer while in Malawi 19% got screened. Screening rates were lowest in India (2%) followed by Bangladesh (7%) [42].

Barriers to implementation of cervical cancer screening programs

Implementation of cervical cancer screening programs in different countries faces different sets of barriers and it is important to evaluate them. Barriers to cervical cancer control efforts are specific to different sociocultural contexts and health systems' preparedness. Countries with organised screening programs will face different challenges compared to countries providing opportunistic screening [43]. Organised programs are better as compared to unorganised programs [8]. Majorly barriers to program implementation will fall in either of the three categories viz-health system specific barriers, health provider specific barriers and patient-specific barriers. Studies exploring barriers to cervical cancer screening program implementation can provide useful information as to how robust a country’s cervical cancer prevention program is. There is a paucity of studies and reports on the implementation of cervical cancer screening programs especially in low-income countries [44].

Maseko et al. [45] assessed health system challenges in cervical cancer screening programs in 14 districts of Malawi. Results showed that challenges in the management of the cervical cancer program at the district level were inadequate service providers who are poorly supervised; lack of basic equipment and stock-outs of basic medical supplies in some health facilities; and inadequate funding of the program. In most health facilities, services providers were not aware of the policy which govern their work and that they did not have standards and guidelines for cervical cancer screening and treatment. Msyamboza et al. [37] documented the success and challenges of the screen and treatment programme (started in 2004) for the first time in Malawi and quoted that due to the high cost of cryotherapy, poor countries could consider cold coagulation for the treatment of precancerous lesions.

In India, the program implementation has been heterogenous across the country since its inception in 2010. Screening rates have been abysmal and most states have not prioritised cancer screening and have not done much beyond setting up Non-Communicable Diseases (NCD) clinics at Community Health Centres (CHCs) and District Hospitals (DHs) [12]. Screening is mainly opportunistic [46]. Deficit in staff and regular availability of drugs, laboratory services, IEC materials, etc., inadequate training and sensitisation regarding the programme hamper program implementation [47]. Dsouza et al. [48] explored barriers to cervical cancer screening through the lens of implementors and beneficiaries and concluded that the participants had low general health concerns, and routine check-ups were considered unimportant. Poor knowledge regarding cervical cancer, the benefits of getting screened, stigma and embarrassment related to the screening procedure were some other hurdles. In certain regions, the geographical inaccessibility of screening services also plays a role in hindering women to participate in cervical cancer screening. A study conducted by Kedar et al. [49] among healthcare providers in Silchar concluded that the lack of human resources and an increased workload on the existing human resource as being the main challenge in the implementation of population-based cancer screening. Other challenges were a lack of clear guidance on the referral pathways, a lack of motivation among health professionals and no awareness of their roles in cancer screening. Bangladesh has a VIA-based program for cervical screening but has a very low coverage of the target population. Bhatla et al. [12] quoted that major gaps found in the program implementation in addition to the very low uptake of screening were wide variation in VIA positivity (1.4% to 12.7%), lack of an information system to track the women referred for colposcopy, and very low compliance with treatment [12,39]. Despite a strong health system, the majority of the primary and secondary health facilities in Bangladesh lack readiness in cervical cancer management in terms of guidelines on diagnosis and treatment, training of staff, and shortage of equipment as quoted by Rakshanda et al. [50].

There are cultural barriers also to screening coverage. A study conducted in Washington by Liang et al. [51] found that women who had more traditional views were significantly less likely to attend regular screening compared to those with less traditional views. Countries such as South Africa have high HIV prevalence and screening uptake is worse in HIV-positive populations [52]. Screening is hampered by clients, providers, and system barriers [34].

Discussion

In this review, we have summarised the screening guidelines and practices in six selected countries based on their economic groups. The health systems and screening guidelines differ from country to country, so making a clear comparison is thus not easy. Countries change and update their screening guidelines over time when new screening tests are developed. Differences have been identified mainly in the age at the commencement of screening and end age, screening intervals and primary screening methods in use. Relatively lower ages for screening have been observed in the USA (21 years) and the UK (25 years). Most countries are recommending commencing screening at the age of 30 years. Similarly, most guidelines recommend terminating screening at 65 years of age. With regard to screening intervals, recommendations rely on the type of screening test being recommended. HPV testing being more sensitive [16] has a longer interval (repeated after three to five years) while cytology usually is recommended to be repeated every one to three years. HPV test is replacing cytology in many European countries as this offers greater protection against cervical cancer and allows longer screening intervals [53]. It is also less dependent on operator expertise [9]. Recommendations based on the status of HIV have been observed in guidelines of countries with high HIV rates - South Africa.

Screening methods adopted by HICs have little relevance for LICs due to multiple factors such as logistics, infrastructure and human resources, factors related to the target population covered, and factors related to program design [9,10]. Cytology-based programs have performed suboptimally in reducing the cervical cancer burden in LMICs due to cost, organisation of the program and poor quality control [8]. Because of this other alternatives have been considered. VIA-based screening has emerged as a low-cost, safe, and effective alternative to cytology screening [54] that has moderate sensitivity and specificity [55] and can be easily administered to a large proportion of targeted women in low-resource settings [54]. It overcomes issues of non-adherence to follow-up visits by providing immediate results in real-time, permitting a single-visit, screen-and-treat approach [54]. A systematic review on the cost-effectiveness of cervical cancer screening methods in LMICs by Mezei et al. [56] concluded that HPV testing and VIA are more cost-effective screening approaches than cytology in LMICs. Self-collected HPV testing was cost-effective when the population coverage gains were higher over other screening methods.

It is important to have screening registries in order to optimise screening programs and improve coverage. Not many countries have such registries. The absence of a central registry of screening records impedes proper monitoring of screening programs and should be adopted by countries that are lacking it. Now that the first cohorts of HPV-vaccinated women are reaching screening age in many countries; such registries can be helpful in evaluating the impact of HPV vaccination on cervical screening performance. Drolet et al. [57] have suggested that screening programs could be de-intensified starting at an older age and with longer screening intervals in settings with high vaccination coverage. However, it is going to be a challenge to personalise screening frequency according to vaccination status as only a fraction of girls must have been vaccinated across the nation.

Screening coverage has been high in USA and UK. This has been supported by Olson et al. [58]. USA doesn’t have a nationwide population-based organised screening program in place [8,22]; nor structured and homogenous monitoring systems. Despite this, screening rates in the USA are among the highest across the globe. This may be attributed to excellent medical/health insurance plans in place in the USA and the delivery of cervical cancer screening is largely the responsibility of health practitioners operating in the context of public/private insurance plans [22]. The UK has had an organised national population-based screening program functional since 1988 and screening data are routinely collected in the national screening registry. Better tracking of programs helps in improving coverage rates. A study by Sankarnarayanan [8] has quoted that organised programs are effective as compared to unorganised programs. Continuous advancements in the type of screening tests lead to the need for future changes and updates in guidelines and practices. Cervical screening programs in Europe are transitioning towards population-based HPV testing [26,27]. A wide range of barriers to screening exists across most LMICs. These could be categorised as health practitioner-specific, individual specific-cultural/traditional/religious and health system and structural barriers to screening. Lack of knowledge and awareness of cervical cancer in general and of screening are the most frequent individual-level barriers [59].

In a nutshell, HICs do not experience many health system barriers due to established screening systems in place. The USA has a good private insurance system and the UK has a well-organised program in which women are invited to get screened. Cultural barriers are common across all countries for cervical cancer screening. South Africa, with decent screening coverage still struggles with cervical cancer control due to high HIV rates. LICs have inadequate infrastructure and trained manpower, inadequate knowledge levels, no screening database, and a lack of invitation systems to successfully implement the programs. Overburdened manpower with other health programs is also a challenge in countries like India because of which cervical screening is not given priority in primary healthcare settings.

Limitations and future prospects

A limitation of this review is that to make the study feasible in the limited time period we have restricted our study to only two countries in each economic group. Nevertheless, we chose countries wisely and the justification for the selection of specific countries in each economic group is presented in the review section. We have obtained an overview of trends in screening coverage, barriers and factors that contributed in spite of program guidelines and in turn how screening guidelines are shaped by resource availability and their optimum utilisation. Adding more countries from diverse geographical backgrounds such as the Middle East will add more value to the study. We adopted a narrative review approach, which may leave scope for potential flaws in our data retrieval method and is a limitation of this study. We have not adopted a systematic approach to select the studies. No specific algorithm was used other than using some keywords relevant to our topic, which could have led to bias during the selection of studies. Despite these flaws, we have tried to give a comprehensive overview of the topic incorporating the screening guidelines for cervical cancer, screening rates, programs and barriers to implementation of the program in the selected countries belonging to different economic groups. The drawbacks could be avoided in future studies by adopting more systematic methods of review of literature avoiding the biases present in this study.

## Conclusions

Pap screening is being replaced by HPV DNA testing in most high and middle-income countries due to its high sensitivity and longer screening intervals. It is also cost-effective. Self-sampling of HPV can further help to improve coverage by increasing opportunities of reaching to women who would otherwise not participate in screening programs. Countries with limited resources recommend VIA-based screening. Countries with high HIV rates may consider updating policies using the recent WHO guidelines. Suitable standard operating procedures should be developed on cervical cancer for each level of health facilities in countries like India and Bangladesh where readiness levels in primary facilities are not sufficient despite national programs being in place for years. Screening coverage could be improved with concerted efforts from the government and NGOs to address the challenges. Low-cost alternatives such as cold coagulation instead of cryotherapy could be considered for the treatment of pre-cancerous lesions in resource-poor settings. LMICs should also consider establishing screening registries in order to track programs and enhance coverage. Further, district-level (de-centralised) research is required to explore the gaps in the health system and the challenges healthcare providers and women experience in the implementation of cervical cancer screening programs. This would lead to a scaling up of national screening efforts.
